# PRINT: A Protein Bioconjugation Method with Exquisite N-terminal Specificity

**DOI:** 10.1038/srep18363

**Published:** 2015-12-17

**Authors:** Surojit Sur, Yuan Qiao, Anja Fries, Robert N. O’Meally, Robert N. Cole, Kenneth W. Kinzler, Bert Vogelstein, Shibin Zhou

**Affiliations:** 1 The Ludwig Center for Cancer Genetics and Howard Hughes Medical Institute at Johns Hopkins Kimmel Cancer Center, Baltimore, MD 21287, USA; 2 Mass Spectrometry and Proteomics Facility, Johns Hopkins University School of Medicine, Baltimore, Maryland 21205, United States

## Abstract

Chemical conjugation is commonly used to enhance the pharmacokinetics, biodistribution, and potency of protein therapeutics, but often leads to non-specific modification or loss of bioactivity. Here, we present a simple, versatile and widely applicable method that allows exquisite N-terminal specific modification of proteins. Combining reversible side-chain blocking and protease mediated cleavage of a commonly used HIS tag appended to a protein, we generate with high yield and purity exquisitely site specific and selective bio-conjugates of TNF-α by using amine reactive NHS ester chemistry. We confirm the N terminal selectivity and specificity using mass spectral analyses and show near complete retention of the biological activity of our model protein both *in vitro* and *in vivo* murine models. We believe that this methodology would be applicable to a variety of potentially therapeutic proteins and the specificity afforded by this technique would allow for rapid generation of novel biologics.

The use of proteins and peptides for therapeutic applications are often compromised by low biological stability, high renal clearance, and non-optimal biodistribution^1^,^2^. Chemical attachment of poly-(ethylene glycol) (PEGylation) is often considered the most effective way to improve these pharmacologic properties by increasing circulation half-life through exclusion from the mononuclear phagocyte system as well as reduced renal clearance, immunogenicity and protease mediated degradation^3^,^4^,^5^,^6^. However, random conjugation results in heterogeneous derivatives with undefined composition and can substantially lower the bioactivity of the modified protein, leading to unpredictable *in vivo* behavior. The same issues apply to conjugations for other purposes, such as the attachment of toxic small molecules to increase the therapeutic efficacy of antibodies.

Site-specific modification of proteins is therefore an attractive approach to circumvent the non-specificity resulting from random conjugation to amines, thiol, or other specific amino acids on proteins. Currently used site-specific strategies exploit rare chemoselective anchors present either naturally or introduced artificially into protein backbones^7^. Amino terminal serines or threonines can be oxidized to aldehydes and targeted using aldehyde-reactive PEG reagents^8^,^9^,^10^,^11^, cysteines have been targeted using thiol-reactive agents^12^,^13^,^14^,^15^, and in a few cases the pKa difference between the α and the ε NH_2_ groups have been used successfully^16^,^17^,^18^. Attempts have even been made to replace all internal lysines to achieve N-terminal selective conjugations^19^,^20^. A recent report has shown that 2-pyridinecarboxaldehydes react with the N terminus of proteins resulting in the formation of imidazolidinone bound conjugates^21^. All of these techniques can be usefully employed, but in view of the ubiquity of this problem and its importance, new ways to site-specifically modify proteins, regardless of the tag used for purification, and with inexpensive, commercially available reagents, are still a high priority.

We developed a novel technique named PRINT (***PR***otect, ***IN***cise ***T***ag) for N-terminal specific bioconjugation of proteins and peptides. In theory, PRINT can be used for selective attachment of any desired entity bearing a nitrogen-reactive functionality. Herein we show that PRINT is able to engineer exclusive N-terminal conjugation of a model protein without altering its biological properties.

## Results

### PRINT Design

**Figure 1:**
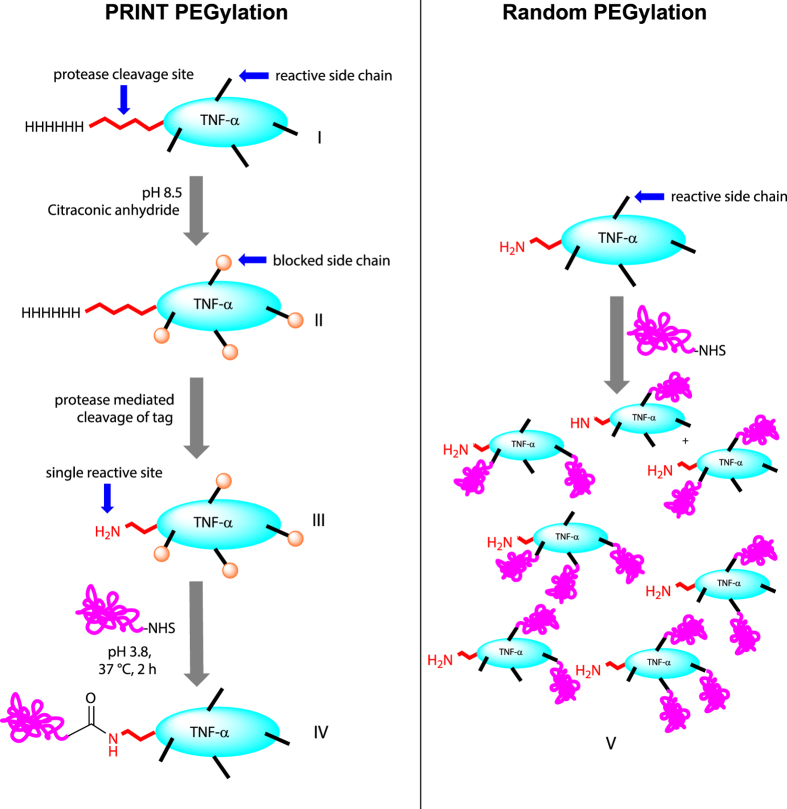
Schematic representation of PRINT PEGylation. The reaction proceeds through blockage of reactive side chains (**II**), followed by protease mediated cleavage to reveal a single reaction site at the N terminus (**III**). Conjugation with NHS ester and subsequent deprotection of side chains leads to N terminal selective and specific conjugate (**IV**). Direct conjugation of the protein using the same NHS ester leads to heterogeneous population of conjugates (**V**).

PRINT was conceptualized to enable N-terminal specific chemical modification, while traditional chemical modification of proteins using amine-reactive NHS ester chemistry leads to heterogeneous and multiple modifications on internal reactive ε NH_2_ groups (Fig. 1). PRINT can be used on any protein that has any desired N-terminal tag (to enhance purification) and any protease cleavage site (to eradicate the tag prior to final purification) (Fig. 1**, I**). The recombinant protein is first treated with an excess of citraconic anhydride to reversibly block all reactive primary amine sites (Fig. 1**, II**). Proteolytic cleavage will then expose *only* a single amine (the α primary amine at the N-terminus) for desired bioconjugation by amine-reactive NHS ester chemistry (Fig. 1**, III**). Lowering of reaction pH will result in removal of the citraconates, leaving N-terminal specific mono PEGylated protein molecules (Fig. 1**, IV**).

As proof of principle, we used Tumor Necrosis Factor-α (TNF-α) to demonstrate the efficiency and specificity of PRINT. A well characterized cytokine, TNF-α has gained attention as a vascular-disrupting agent specific to tumors^22^,^23^,^24^,^25^. However, TNF-α, like many other potential therapeutic proteins, suffers from inherent instability and short biological half-life, and exhibits toxic side effects at therapeutic concentrations in both small animals and human patients. Altering its pharmacokinetic profile by PEGylation has been shown to enhance its stability and bioavailability^26^,^27^,^28^, and to mitigate its toxicity^19^,^20^,^29^,^30^,^31^. In this study, we used a recombinant single-chain form consisting of three head-to-tail copies of the monomer, as this has been shown to enhance formation of an active protein from bacteria^32^.

### PRINT using scTNF-α as a model protein

**Figure 2:**
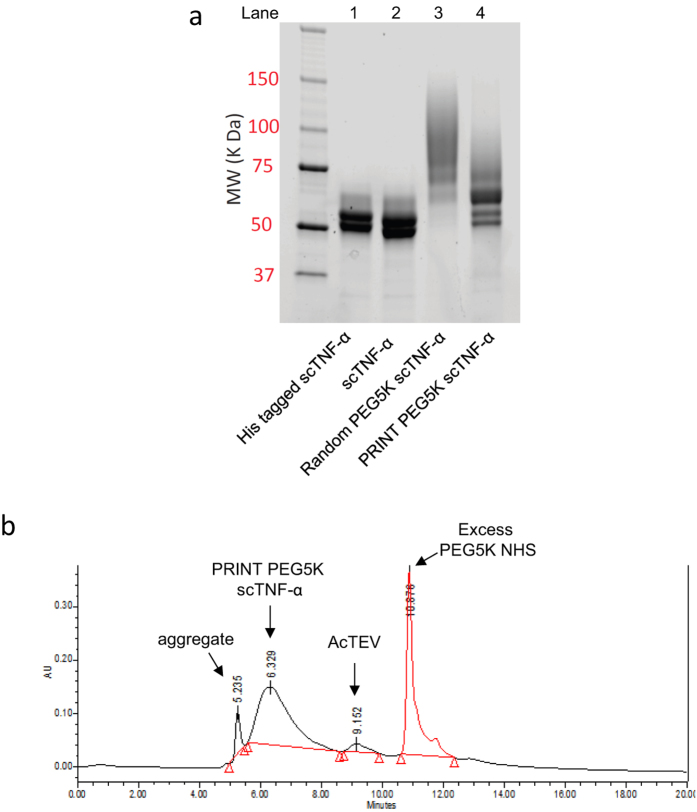
(**a**) SDS-PAGE characterization of scTNF-α derivatives: Lanes (left to right): Protein standard; Lane 1, His tagged scTNF-α (**I**); Lane 2, cleaved scTNF-α (scTNF-α) (**II**); Lane 3, directly PEGylated PEG5K scTNF-α (random PEG5K scTNF-α) (**V**); Lane 4, PRINT PEG5K scTNF-α (**IV**). (**b**) SEC HPLC of PRINT PEG5K scTNF-α.

A recombinant single-chain TNF-α (scTNF-α) containing a His-tag and TEV protease cleavage site was designed based on a published sequence^32^. After affinity purification through a nickel-nitrilotriacetic acid (Ni-NTA) column, the His-tagged scTNF-α was treated with a 1000-fold molar excess of citraconic anhydride. Excess reagent was removed by dialysis and the citraconylated protein was subjected to overnight digestion with AcTEV protease. After complete proteolytic cleavage of the His-tag, NHS ester of PEG-5000 (PEG5K) was added and the mixture allowed to shake at room temperature for 30 minutes. Excess reagent was then removed and pH adjusted to 3.8 for deprotection of side chains. These treatments yielded a major N-terminal mono PEGylated species (Fig. 2a**, Lane 4**). In comparison, a traditional PEGylation method without PRINT generated multiple species of various lengths, indicating the expected large and variable numbers of internal reactive NH_2_ groups getting PEGylated (Fig. 2a**, Lane 3**). A control PEGylation on citraconylated scTNF prior to removal of its His-tag yielded no PEGylated products (Supplementary Fig. 1**, Lane 3**), demonstrating complete blocking of the reactive α and ε NH_2_ groups present on the protein. Size exclusion high-performance liquid chromatography (HPLC) analysis of PRINT PEGylated scTNF-α revealed the formation of a major mono PEGylated product (Fig. 2b). A small peak at retention time 5.935 min is noticeable, likely representing protein aggregates. The N-termini of protein molecules buried inside the aggregates could be inaccessible to PEG NHS, resulting in the small amount of non-PEGylated protein shown on SDS-PAGE (Fig. 2a**, Lane 4**). Because this process was simple and effective, several other conjugates of scTNF-α were able to be synthesized for biological evaluation starting from small amounts of purified proteins (Supplementary Table 1).

### PRINT provides N terminal selectivity

To elucidate the exact location of the conjugation, we replaced the reactive PEG5K with fluorescein NHS (Fl) ester, a smaller adduct with a known exact mass of 358 Da (Supplementary Fig. 1a**, lane 4 and** Supplementary Fig. 1b). Proteolytic cleavage of PRINT flourescein scTNF-α with trypsin followed by mass spectral analysis confirmed the presence of a single fluorescein molecule at the N-terminal serine (Supplementary Fig. 2). No other peptide fragment containing fluorescein was detected (Supplementary Table 2), suggesting an exquisite N-terminal selectivity and specificity of the reaction.

### PRINT retains bioactivity of scTNF-α

**Figure 3:**
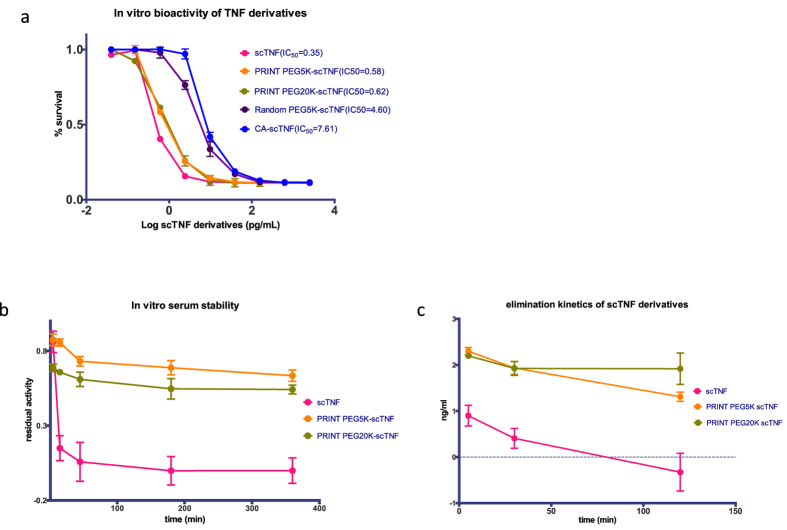
(**a**) *In vitro* bioactivity of scTNF-α derivatives in L929 cells. (**b**) *In vitro* serum stability and residual activity of scTNF-α, PRINT PEG5K and PRINT PEG20K scTNF-α. (**c**) *In vivo* clearance of scTNF-α and its PEGylated derivatives.

To assess bioactivity of the PRINT PEGylated scTNF-α, we performed a cytotoxicity assay using L929 cells that express TNFR1, the receptor mediating TNF-α induced cytotoxicity. Unmodified scTNF-α and scTNF-α that had been PRINT-PEGylated with PEG5K or PEG-20000 (PEG20K) all showed similar cytotoxic activity against L929 cells, with EC_50_ of 0.35, 0.58 and 0.62 pg/mL, respectively (Fig. 3a). In contrast, randomly PEGylated scTNF-α suffered more than ten-fold loss of activity, resulting in an EC_50_ of 4.6 pg/mL. Similarly, global blocking of lysine side chains by citraconylation dramatically reduced (EC_50_ = 7.6 pg/mL) its bioactivity, thereby providing biological confirmation that the citraconate groups had been removed.

### PRINT reduces scTNF-α toxicity

To assess toxicity *in vivo*, wild-type mouse TNF-α, unmodified scTNF-α and PRINT- PEGylated (PEG5K) scTNF-α were intravenously injected at various doses into BALB/c mice bearing large subcutaneous CT26 tumors. Mice bearing large tumors were used because they are more sensitive to TNF-α induced toxicity than non-tumor-bearing mice (ref here). At a dose of 150 μg/kg all 10 animals treated with mouse wt TNF-α or unmodified scTNF-α died within 24 hours. In contrast, none of the 10 animals treated with PRINT PEGylated (PEG5K or PEG20K) scTNF-α at the same or higher doses showed any adverse event (Supplementary Fig. 2).

### PRINT enhances stability and circulation half-life of scTNF-α

Finally, we evaluated stability of the unmodified scTNF-α, PRINT PEGylated (PEG5K) scTNF-α, and PRINT-PEGylated (PEG20K) scTNF-α. We first assessed their serum stability *ex vivo*. Both PRINT-PEGylated scTNF-α molecules showed greatly improved stability compared to the unmodified scTNF-α (Fig. 3b). We then intravenously injected the TNF-α preparations into non-tumor-bearing healthy BALB/c mice and collected blood samples at various time points. The unmodified scTNF-α showed a rapid clearance from the bloodstream, as assessed by enzyme-linked immunosorbent assay (ELISA), and was undetectable at 2 h (Fig. 3c). In contrast, the two PRINT PEGylated scTNF-α molecules showed substantially higher persistence in the bloodstream and low clearance rate.

## Discussion

In conclusion, we have demonstrated that the side chain protection before cleavage of the tag efficiently blocked all reactions at the side chains (Supplementary Fig. 1a**, Lane 3**). The single product formed after protease-mediated tag removal and N-terminal conjugation suggests exquisite selectivity and specificity in contrast to conventional reaction using the same NHS reagent (compare Fig. 2a**, Lane 4,** Supplementary Fig. 1a**, Lanes 4, 5 and 7** with Fig. 1a**, Lane 3 and** Supplementary Fig. 1a**, Lane 6**), which was further confirmed by mass spectrometric analyses. Subsequent de-blocking generated an N-terminal protected TNF-α molecule with enhanced serum stability, superior pharmacokinetic properties, and reduced systemic toxicity (Fig. 3b,c and Supplementary Fig. 3). Importantly, N-terminal protection by PRINT did not affect the bioactivity of TNF-α (Fig. 3a).

As noted in the introduction, existing site-selective bioconjugation approaches are either specific to amino acid tags^7^,^8^,^9^,^10^,^11^,^33^,^34^ or involve substantial non-trivial chemical^18^,^21^ or biotechnological manipulations^19^,^20^ to synthesize a desired bioconjugate. In contrast, PRINT employs ubiquitously used recombinant DNA techniques and easily acquired commercial reagents to generate exquisite N-terminal selective protection. In this study, we used TNF-α as an example to show that PRINT is a robust, reproducible and mild strategy which is able to target the α-amine and provide N-terminal specific protection to proteins or peptides that suffer from similar issues. In principle, PRINT can be used to generate a variety of N-terminal conjugates using NHS ester chemistry on any recombinant protein or peptide bearing a cleavable purification tag. We believe that this approach is strongly orthogonal to current methods and will be applicable to many biotherapeutics and bioprobes that are currently being designed to treat cancer or other diseases.

## Methods

### General Materials and Methods

Citraconic anhydride(Sigma), Sodium phosphate dibasic and monobasic (Sigma), mPEG 5K NHS ester (NANOCS), mPEG 20K NHS ester (NANOCS), Fluorescein NHS ester (NANOCS) and AcTEV (Life Technologies) were obtained from commercial sources and used as is. Single chain TNF-α (**scTNF)** was designed according to a published sequence and the recombinant protein was produced by GeneArt in HEK293 mammalian expression system. All animal experiments were designed in accordance with the National Institute of Health’s *Guide for the Care and Use of Laboratory Animals* and were approved by The Johns Hopkins University’s Institutional Animal Care and Use Committee.

### Direct Conjugation

scTNF-α (1 mg/ml in PBS) was treated with PEG NHS ester (1 mg) for 1 h at room temperature and excess reagents were removed by dialyses. The recovered product was analyzed and quantitation by done by SDS-PAGE and used as such for *in vitro* and *in vivo* animal experiments.

### PRINT Conjugation

scTNF-α (1 mg/ml in 200 mM phosphate buffer at pH 8.5) was treated with citraconic anhydride (3ul/100 ug protein) at room temperature^35^ for 5 minutes. The mixture was then dialyzed against 500 ml phosphate buffer (200 mM, pH 8.5) for 8 hours. AcTEV (5 ul/100 ug protein) was then added and the mixture allowed to shake gently at room temp overnight. PEG NHS esters (20–50×) was then added and the mixture allowed to incubate for 1 hour at room temp. The mixture was then dialyzed against 1L acetate buffer (200 mM, pH 3.8) at room temperature overnight followed by buffer exchange against PBS 1L twice. AcTEV was then removed from the product by NiNTA spin columns following manufacturer instructions. The products were then analyzed for purity and quantitated for protein content by SDS-PAGE and used as such for *in vitro* and *in vivo* animal experiments.

### SDS-PAGE and protein quantitation

Protein samples were analyzed for purity using Biorad Stain Free TGX precast gels. In brief, 3 ul of protein samples was diluted with deionized water (6 ul) followed by 3 ul of Laemlli buffer (4X). After electrophoresis, gels was developed using a Biorad ChemiDoc MP imaging system and quantitation was performed using Imagelab software against standards containing known quantity of scTNF-α.

### Mass Spectral Analyses by Liquid Chromatography-Tandem Mass Spectrometry (LC-MS)

For Mass Spectral analyses, the product was further purified by Size Exclusion chromatography using a Phenomenex BioSep-SEC-s2000 (300 × 7.8 mm) column. Samples of 100 ul were injected, and separations carried out using PBS (pH 7.4) as the mobile phase at ambient temperature and flow rate of 1.00 ml/min on a Waters D600 HPLC system using Absorbance at 220 nm. Protein samples from either gel bands or size-exclusion chromatography were proteolyzed with trypsin as described previously. Digested peptides were extracted and subjected to vacuum drying in a Speedvac followed by reconstitution in 5 μL of 2% acetonitrile/0.1% formic acid for further analysis by liquid chromatography/tandem mass spectrometry (LC–MS/MS) using LTQ Orbitrap Velos (2) MS (Thermo Fisher Scientific). For data analysis the data was submitted for a Sequest search using Proteome Discoverer v 1.3 (Thermo Fisher Scientific) against the constructed sequence database. The Fluorescein modification of 358.040 was set to variable at K and Y and static for the N-terminus.

### *In vitro* cytotoxicity assay

Conjugated proteins were assessed for bioactivity using previously described TNF-α induced killing of L929 cells. L929 cells (Sigma # **85011425**) were plated at density of 3.5 × 10^5^ cells per well in 96 well plates and incubated overnight at 37 °C in a humidified incubator. A 4 fold dilution series for each sample was created starting at 2.5 ng/mL. Cells were then treated with 50 ul of TNF derivatives at each concentration along with 50 ul Actinomycin D (4 ug/ml) and allowed to incubate 24 h. Potency of the TNF-α derivatives was assayed using cell proliferation reagent WST-1 (Roche Lifesciences) following manufacturers protocol.

### *In vitro* stability assay

scTNF-α and its PRINT Pegylated derivatives were incubated with mouse serum at 37 °C for 24 h and aliquots were collected at various time points (5, 15, 45 min, 1.5, 3, 6 and 12 h) and frozen immediately. Once all desired time points were collected, the samples were thawed and analyzed for residual bioactivity using the L929 cytotoxicity assay.

### *In vivo* pharmacokinetics

The pharmacokinetic characteristics of scTNF-α derivatives was investigated in mice following intravenous (i.v.) administration. Healthy female BALB/c mice were randomly divided to 3 groups (n = 3) and each group was administered 150 μg/kg (protein base) of TNF-α derivatives Blood samples were collected at different time points (5, 30 min and 2h) after i.v. injection, and plasma were obtained by centrifugation and stored at −70 °C until required for the assay. scTNF-α concentrations in mice plasma were measured and quantitated using a commercial TNF ELISA kit (R & D Systems) and a dilution series of known amounts of scTNF-α as standard.

## Additional Information

**How to cite this article**: Sur, S. *et al.* PRINT: A Protein Bioconjugation Method with Exquisite N-terminal Specificity. *Sci. Rep.* **5**, 18363; doi: 10.1038/srep18363 (2015).

## Supplementary Material

Supplementary Information
