# Supplementary material for: PRINT: A Protein Bioconjugation Method with Exquisite N-terminal Specificity
Source: Sci Rep. 2015 Dec 17;5:18363. doi: 10.1038/srep18363 (PMC4683619; doi:10.1038/srep18363)
Supplement: Supplementary Information [file srep18363-s1.pdf]

PRINT: A Protein Bioconjugation Method with Exquisite N-terminal Specificity.

Surojit Sur[1], Yuan Qiao[1], Anja Fries[1], Robert N. O'Meally[2], Robert N. Cole[2], Kenneth W. Kinzler[1], Bert Vogelstein[1] and Shibin Zhou[1]

[1] The Ludwig Center for Cancer Genetics and Howard Hughes Medical Institute at Johns Hopkins Kimmel Cancer Center, Baltimore, MD 21287, USA.
[2] Mass Spectrometry and Proteomics Facility, Johns Hopkins University School of Medicine, Baltimore, Maryland 21205, United States
Correspondence should be addressed to S.S. (ssur1@jhmi.edu), B.V. (vogelbe@jhmi.edu) or S.Z. (sbzhou@jhmi.edu)

Supplementary Figure Legends

Supplementary Table 1: List of scTNF-α derivatives synthesized by PRINT

Supplementary Figure 1: **a)** SDS PAGE characterization: Lanes from left to right: protein standard, Lane 1, His tagged scTNF-α; Lane 2, CA-protected protease cleaved scTNF-α; Lane 3, CA protected His-tagged scTNF-α treated with 1000 x PEG5K NHS (His-tagged blocked protein is unable to react with PEG5K NHS- negative control); Lane 4, PRINT Fluorescein scTNF-α; Lane 5, PRINT PEG5K scTNF-α; Lane 6, random PEG5K scTNF-α; Lane 7, PRINT PEG20K scTNF-α. **b)** Overlay of SEC-HPLC of Fluorescein scTNF at two wavelengths 220 (black) nm and 482 nm (green).

Supplementary Figure 2: MS/MS analyses of the tryptic peptide GRSSQNSSDKPVAH modified with Fluorescein: Fluorescein NHS ester was used instead of PEG5K NHS, allowing us to identify peptide fragments labelled with an exact mass of 358.04 (arrows). The b ions are shown in red, y ions are shown in blue. Fragment ion masses were consistent with modification at the N-terminus and no other peptides with a mass increase of 358.04 were detected.

Supplementary Table 2: List of peptides with additional mass of 358, detected for tryptic fragment GRSSQNSSDKPVAH.

Supplementary Figure 3: Acute toxicity of wt TNF-α, scTNF-α and its derivatives in BALB/c mice harboring CT26 tumors.10 mice in each study arm were injected with a single i.v. dose of various forms of TNF-α at different doses. The results were scored by surviving mice at the end of a 24 h time period.

	Conjugating reagent
1	PEG 20K NHS
2	Folate PEG 3400K NHS
3	Fluorescein NHS
4	TMS PEG NHS
5	AMCA NHS
6	β-Cyclodextrin caproate NHS

Sur *et al.* Supplementary Table 1

a

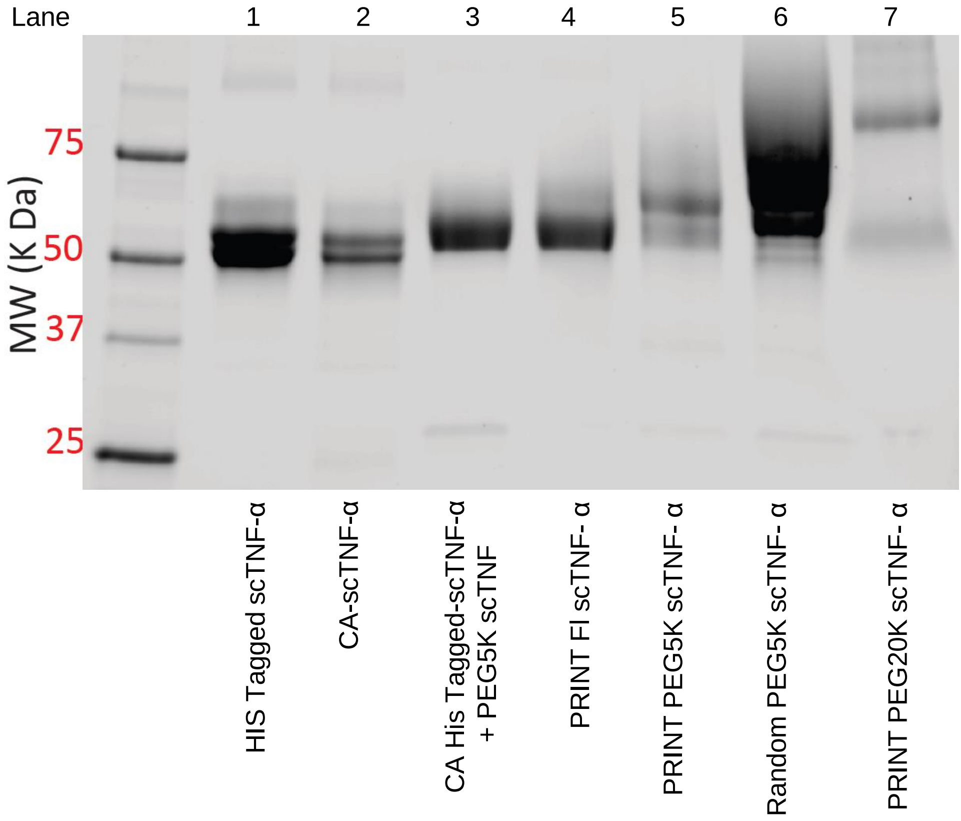

b

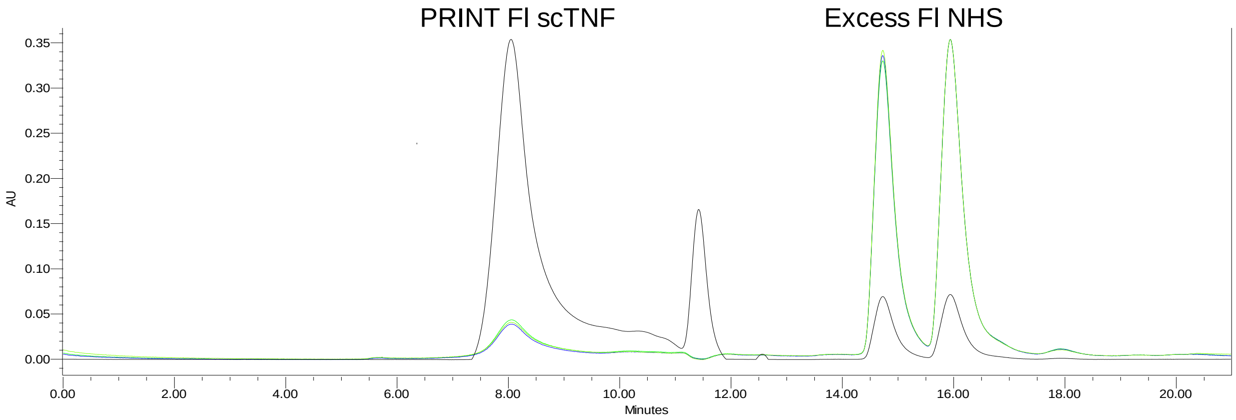

Sur *et al.* Supplementary Fig. 1

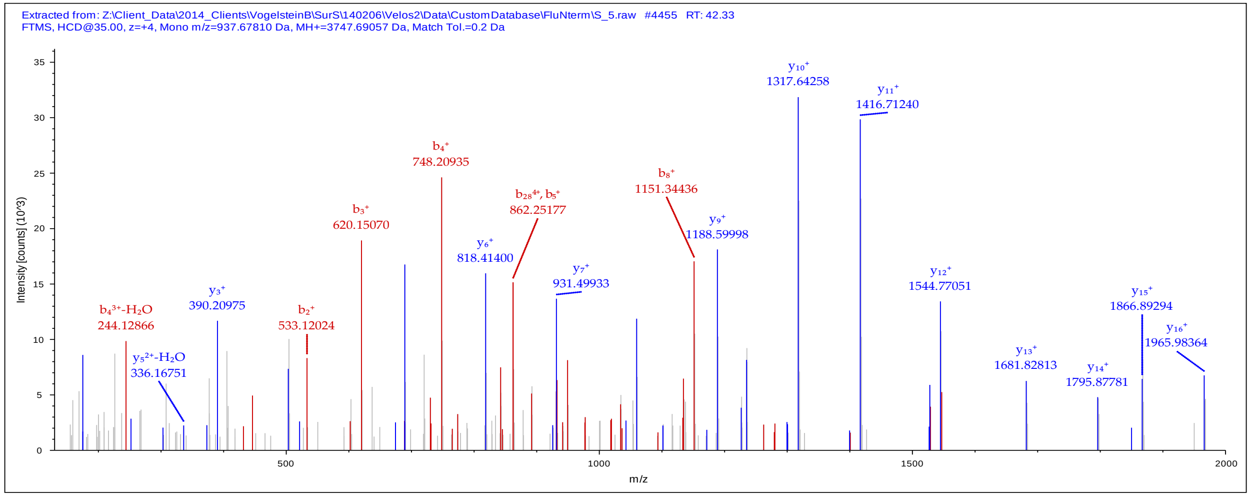

#1	b^{+}	b^{2+}	b^{3+}	b^{4+}	Seq.	y^{+}	y^{2+}	y^{3+}	y^{4+}
1	446.07931	223.54329	149.36462	112.27528	S-FluScn				
2	533.11134	267.05931	178.37530	134.03329	S	3302.61498	1651.81113	1101.54318	826.40920
3	620.14337	310.57532	207.38597	155.79130	S	3215.58295	1608.29511	1072.53250	804.65120
4	748.20195	374.60461	250.07217	187.80594	Q	3128.55092	1564.77910	1043.52182	782.89319
5	862.24488	431.62608	288.08648	216.31668	N	3000.49234	1500.74981	1000.83563	750.87854
6	949.27691	475.14209	317.09715	238.07468	S	2886.44941	1443.72834	962.82132	722.36781
7	1036.30894	518.65811	346.10783	259.83269	S	2799.41738	1400.21233	933.81064	700.60980
8	1151.33589	576.17158	384.45015	288.58943	D	2712.38535	1356.69631	904.79997	678.85180
9	1279.43086	640.21907	427.14847	320.61317	K	2597.35840	1299.18284	866.45765	650.09506
10	1376.48363	688.74545	459.49939	344.87636	P	2469.26343	1235.13535	823.75933	618.07132
11	1475.55205	738.27966	492.52220	369.64347	V	2372.21066	1186.60897	791.40840	593.80812
12	1546.58917	773.79822	516.20124	387.40275	A	2273.14224	1137.07476	758.38560	569.04102
13	1683.64808	842.32768	561.88754	421.66748	H	2202.10512	1101.55620	734.70656	551.28174
14	1782.71650	891.86189	594.91035	446.43458	V	2065.04621	1033.02674	689.02025	517.01701
15	1881.78492	941.39610	627.93316	471.20169	V	1965.97779	983.49253	655.99745	492.24991
16	1952.82204	976.91466	651.61220	488.96097	A	1866.90937	933.95832	622.97464	467.48280
17	2066.86497	1033.93612	689.62651	517.47170	N	1795.87225	898.43976	599.29560	449.72352
18	2203.92388	1102.46558	735.31281	551.73643	H	1681.82932	841.41830	561.28129	421.21279
19	2331.98246	1166.49487	777.99900	583.75107	Q	1544.77041	772.88884	515.59499	386.94806
20	2431.05088	1216.02908	811.02181	608.51818	V	1416.71183	708.85955	472.90879	354.93342
21	2560.09348	1280.55038	854.03601	640.77883	E	1317.64341	659.32534	439.88599	330.16631
22	2689.13608	1345.07168	897.05021	673.03948	E	1188.60081	594.80404	396.87179	297.90566
23	2817.19466	1409.10097	939.73640	705.05412	Q	1059.55821	530.28274	353.85759	265.64501
24	2930.27873	1465.64300	977.43109	733.32514	L	931.49963	466.25345	311.17139	233.63037
25	3059.32133	1530.16430	1020.44529	765.58579	E	818.41556	409.71142	273.47670	205.35935
26	3245.40065	1623.20396	1082.47173	812.10562	W	689.37296	345.19012	230.46250	173.09870
27	3358.48472	1679.74600	1120.16642	840.37664	L	503.29364	252.15046	168.43606	126.57887
28	3445.51675	1723.26201	1149.17710	862.13464	S	390.20957	195.60842	130.74137	98.30785
29	3573.57533	1787.29130	1191.86329	894.14929	Q	303.17754	152.09241	101.73070	76.54984
30					R	175.11896	88.06312	59.04450	44.53520

Sur *et al.* Supplementary Fig. 2 and Table 2

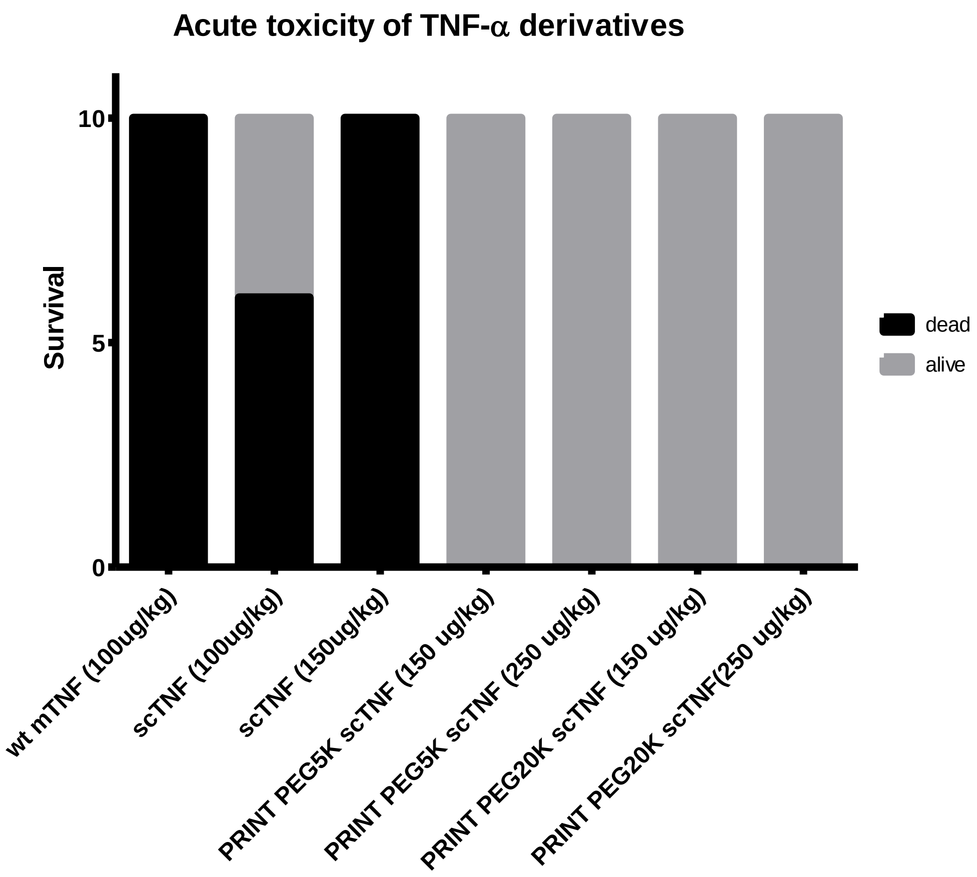

Sur *et al.* Supplementary Fig 3
